# Global Transcriptome Analysis of Brown Adipose Tissue of Diet-Induced Obese Mice

**DOI:** 10.3390/ijms19041095

**Published:** 2018-04-06

**Authors:** Jingyi Cao, Qi Zhu, Lin Liu, Bradley J. Glazier, Benjamin C. Hinkel, Chun Liang, Haifei Shi

**Affiliations:** 1 Program of Physiology and Neuroscience, Department of Biology, Miami University, Oxford, OH 45056, USA; caoj2@miamioh.edu (J.C.); zhuq2@miamioh.edu (Q.Z.); glazieb2@miamioh.edu (B.J.G.); hinkelbc@miamioh.edu (B.C.H.); 2 Program of Bioinformatics, Department of Biology, Miami University, Oxford, OH 45056, USA; liul2@miamioh.edu (L.L.); liangc@miamioh.edu (C.L.)

**Keywords:** high-fat diet, RNA sequencing, brown adipose tissue, muscle system process, inflammation, calcium signaling, ion transport

## Abstract

Consumption of a high-fat diet (HFD) promotes the development of obesity, a disease resulting from an imbalance between energy intake and energy expenditure. Brown adipose tissue (BAT) has thermogenic capacity that burns calories to produce heat, and it is a potential target for the treatment and prevention of obesity. There is limited information regarding the impact of HFD on the BAT transcriptome. We hypothesized that HFD-induced obesity would lead to transcriptional regulation of BAT genes. RNA sequencing was used to generate global transcriptome profiles from BAT of lean mice fed with a low-fat diet (LFD) and obese mice fed with a HFD. Gene Ontology (GO) analysis identified increased expression of genes involved in biological processes (BP) related to immune responses, which enhanced molecular function (MF) in chemokine activity; decreased expression of genes involved in BP related to ion transport and muscle structure development, which reduced MF in channel and transporter activity and structural binding. Kyoto Encyclopedia of Genes and Genomes (KEGG) functional pathway analysis indicated that pathways associated with innate immunity were enhanced by HFD, while pathways associated with muscle contraction and calcium signaling were suppressed by HFD. Collectively, these results suggest that diet-induced obesity changes transcriptomic signatures of BAT, leading to dysfunction involving inflammation, calcium signaling, ion transport, and cell structural development.

## 1. Introduction

Obesity, characterized by increased energy storage within the body, has become one of the most noticeable epidemics worldwide, with over 80 million children and adults in the U.S. being obese [1]. Obesity is closely associated with other disease processes, including type 2 diabetes, hyperlipidemia, cardiovascular disorders, non-alcoholic hepatic steatosis, and certain types of cancer [2]. The high-fat content in typical Western diets is one of the most important environmental factors leading to obesity [3]. Increased research attention is focused on the regulation of physiological and pathological functions of adipose tissues by high-fat diets.

White adipose tissue (WAT) is classically considered as a main energy storage reservoir where energy is accumulated within the body in the form of lipids; whereas brown adipose tissue (BAT) has the remarkable ability to dissipate excess energy as heat in a process known as adaptive thermogenesis [4,5,6]. The discovery of inducible BAT in adult humans renewed interest of involving BAT in metabolic regulation [7,8,9,10]. Obese subjects, including humans, have reduced BAT thermogenic activity in response to dietary fat [11,12]. In contrast, functional BAT improves energy homeostasis, plasma lipid profile, and insulin sensitivity in humans [9,13,14]. It has been widely accepted that BAT mitochondrial uncoupling protein 1 (UCP1) [4] and fibroblast growth factor-21 (FGF21) [15,16] play critical roles in non-shivering thermogenesis to maintain body temperature. Recently, increasing evidence has suggested that fatty acid oxidation occurs independently of the expression of thermogenic gene *Ucp1* in BAT [17]. Interestingly, mice that lack UCP1 and FGF21 maintain normal body temperature and thermogenesis during cold exposure [18], suggesting alternative pathways involved in BAT thermogenesis for further exploration. The developmental consequence of brown adipocytes arising from common progenitor mesodermal stem cell lineage is determined during embryonic development. This progenitor cell lineage could give rise to fibroblast-like brown adipogenic progenitors, a process regulated by morphogenic genes for developmental regulation such as *Bmp8b*, *Tgfb*, and *Fgf* [19]. Thus, elucidating underlying mechanisms involved in both inducing brown adipocyte differentiation and maintaining BAT function would provide invaluable research avenue for developing new strategies to counter obesity, diabetes, and hypertriglyceridemia [8,20].

We hypothesized that high-fat diet (HFD)-induced obesity would lead to transcriptional regulation of BAT genes. Diet-induced obesity affects gene expression in many metabolic tissues, such as subcutaneous, gonadal, and visceral WAT [21,22]; liver [23,24,25]; colonic tissue [26], etc. Research on BAT gene expression change in diet-induced obesity is relatively scarce. When we searched PubMed using ((brown adipose tissue) AND high fat diet) AND ((((microarray) OR RNA sequencing) OR RNA seq) OR sequencing) on 12 February 2018, 26 hits were returned (Table S1). Among them, 4 studies identified differential expression of genes in BAT of HFD-induced obese mice [27,28,29,30], and 2 studies identified genes in BAT of obese rats [31,32], and all these six studies used microarray and PCR techniques to examine differential expression of genes in BAT. RNA sequencing (RNA-Seq) is a newly developed sequencing-based method that examines expression of the global transcriptome. Compared to microarray, RNA-Seq has many notable advantages [33,34]. One advantage is that RNA-Seq has a broader dynamic range and higher sensitivity to detect low abundance transcripts than microarray, allowing for the detection of more differentially expressed genes with higher fold-change [33,34]. Another advantage is that RNA-Seq identifies not only known genes, but also unknown transcripts, allowing for the identification of unknown genes. Additionally, RNA-Seq is devoid of certain technical issues inherent to microarray probe performance, such as cross-hybridization, non-specific hybridization, and probe redundancy and annotation.

In this study, we used RNA-Seq to generate comprehensive and comparable global transcriptome profiles and identified differentially expressed genes in BAT of lean mice fed a standard low-fat diet (LFD) and obese mice fed a HFD for four weeks, a time point that mice become apparently obese and insulin resistance and inflammation are initiated [35,36,37]. BAT transcriptome changes detected in this study are, therefore, due to diet-induced obesity along with insulin resistance and inflammation, but are unlikely to be the consequence of cardiovascular dysfunction or hyperlipidemia that are developed following a prolonged period of HFD feeding. The transcriptome analysis revealed many differentially expressed genes in BAT between lean and obese mice. The top downregulated genes were primarily involved in muscle development, muscle system process, regulation of ion transport and neurotransmitter secretion. The top upregulated genes were mostly involved in alterations in inflammation, such as chemotaxis of leukocytes, macrophages, and monocytes, as well as T cell-mediated immunity. The distinct BAT gene expression profiles between lean and obese mice underlie potential dysfunction of BAT that contributes to the development of diet-induced obesity.

## 2. Results

### 2.1. Body Mass, Body Composition, and BAT Mass

At the initiation of feeding with different diets, LFD- and HFD-fed groups had similar body mass (LFD: 21.49 ± 0.35 g; HFD: 21.27 ± 0.33 g; *P* > 0.05), fat mass (LFD: 2.05 ± 0.15 g; HFD: 1.94 ± 0.14 g; *P* > 0.05), and lean mass (LFD: 17.72 ± 0.25 g; HFD: 17.70 ± 0.20 g; *P* > 0.05). After 4 weeks of feeding, HFD-fed group had significantly greater body mass (LFD: 25.77 ± 1.10 g; HFD: 30.31 ± 1.28 g; *P* < 0.05), fat mass (LFD: 2.77 ± 0.26 g; HFD: 8.36 ± 1.13 g; *P* < 0.05), and BAT mass (LFD: 82.82 ± 8.70 mg; HD: 174.84 ± 17.58 mg; *P* < 0.05) than LFD-fed group, whereas their lean mass was similar (LFD: 20.32 ± 1.10 g; HFD: 20.20 ± 1.33 g; *P* > 0.05). The average fat mass of the HFD-fed mice was greater than the average fat mass + 3 standard deviation of the LFD group, and thus HFD group mice were considered obese [38].

### 2.2. Transcriptome Analysis

Transcriptome-wide expression analysis was performed using RNA-Seq, which resulted in about 30 million raw reads of 75 bases each, with >98% of high quality reads with Phred quality score (Q score) > 30. Q30 is Illumina’s quality score that provides 99.9% accuracy [39]. A summary of the reads for each sample is presented in Table 1, including total raw reads, high-quality reads, and adapter removed reads.

**Figure 1:**
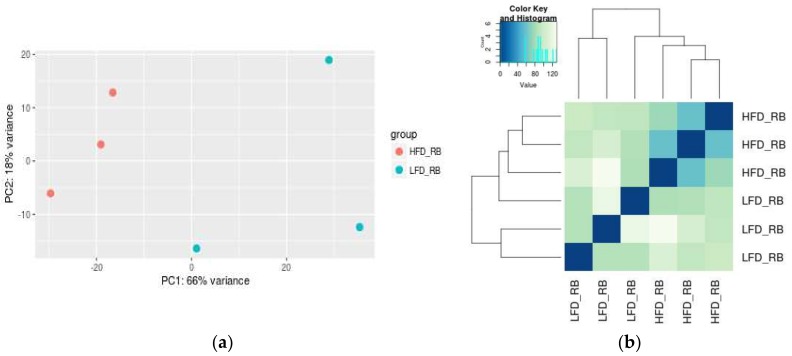
Clusters of the differentially expressed genes in the brown adipose tissues (BAT) of the low-fat diet (LFD)-fed lean mice and high-fat diet (HFD)-fed obese mice. (**a**) Principal component analysis (PCA) of RNA sequencing (RNA-Seq) data revealed distinct gene expression in BAT between LFD- and HFD-fed mice. Each dot represents a sequencing library and consists of normalized read counts for high quality reads. (**b**) Euclidean distance analysis showed clustering of gene expression in BAT of LFD- and HFD-fed mice.

The transcriptome analysis of BAT from lean and obese mice revealed top 357 differentially expressed genes among 29,856 identified genes, with 265 genes downregulated and 92 genes upregulated. By performing the principle component analysis (PCA; Figure 1a) and analyzing the Euclidean distance among samples (Figure 1b) based on the significant deferentially expressed genes, the BAT of LFD- and HFD-fed mice were clustered into two distinct subgroups using triple biological replicates.

**Figure 2:**
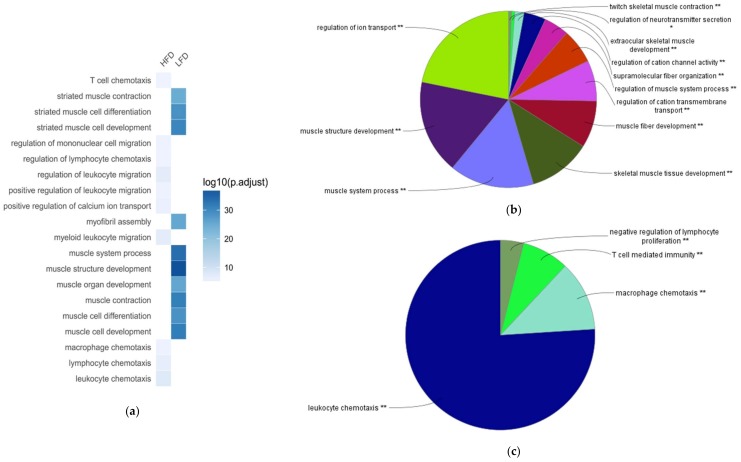
Comparing top Gene Ontology (GO) biological process (BP) terms associated with high-fat diet (HFD)-responsive genes in brown adipose tissue (BAT). Enrichment analysis was performed using differentially expressed genes in BAT between HFD and low-fat diet (LFD) groups. (**a**) Heat-map showing top GO BP annotation terms in the BAT between HFD and LFD groups. (**b**) GO BP terms associated with downregulated genes in BAT of HFD-fed obese mice. (**c**) GO BP terms associated with upregulated genes in BAT of HFD-fed obese mice.

**Figure 3:**
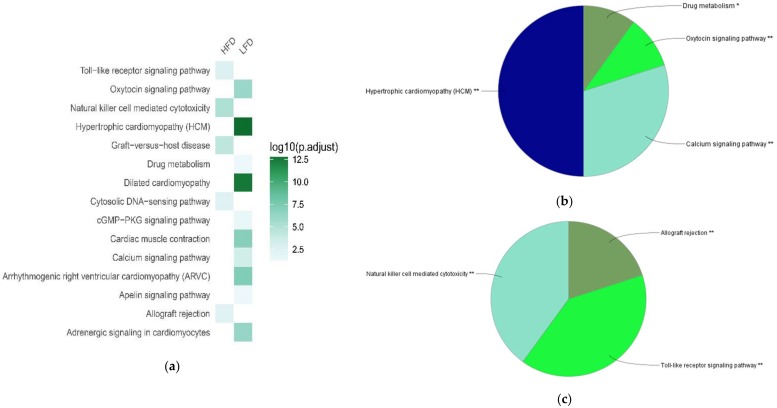
Comparing top Gene Ontology (GO) Kyoto Encyclopedia of Genes and Genomes (KEGG) terms associated with high-fat diet (HFD)-responsive genes in brown adipose tissue (BAT). Enrichment analysis was performed using differentially expressed genes in BAT between HFD and low-fat diet (LFD) groups. (**a**) Heat-map showing top GO KEGG annotation terms in BAT between HFD and LFD groups. (**b**) GO KEGG terms associated with downregulated genes in BAT of HFD-fed obese mice. (**c**) GO KEGG terms associated with upregulated genes in BAT of HFD-fed obese mice.

**Figure 4:**
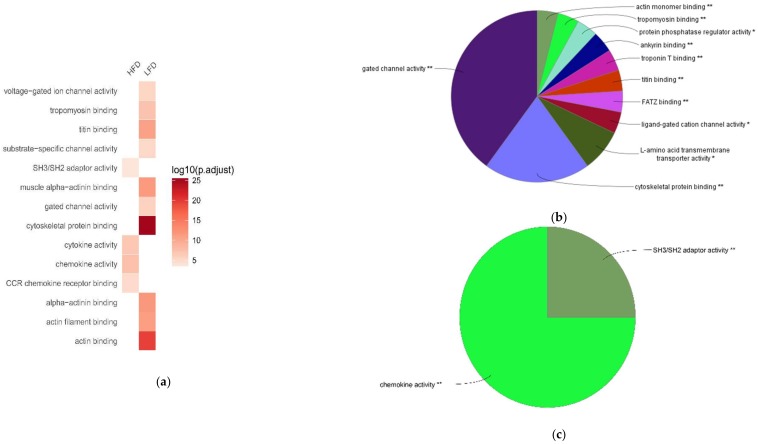
Comparing top Gene Ontology (GO) molecular function (MF) terms associated with high-fat diet (HFD)-responsive genes in brown adipose tissue (BAT). Enrichment analysis was performed using differentially expressed genes in BAT between HFD and low-fat diet (LFD) groups. (**a**) Heat-map showing top GO MF annotation terms in the BAT between HFD and LFD groups. (**b**) GO MF terms associated with downregulated genes in BAT of HFD-fed obese mice. (**c**) GO MF terms associated with upregulated genes in BAT of HFD-fed obese mice.

Next, we compared the biological characteristics of these differentially expressed genes in BAT between LFD-fed lean mice and HFD-induced obese mice, using Gene Ontology (GO) enrichment analysis. The areas of the pie charts in Figure 2, Figure 3 and Figure 4 indicate the % of identified genes from GO analyses that are associated with individual identified pathways. The majority of the downregulated genes in BAT of HFD-fed obese mice were associated with biological processes (BP) annotation terms related to muscle structure and tissue development, ion transport and ion channel activity, muscle system process and contraction, and neurotransmitter secretion (Figure 2a,b; Table S2). These downregulated genes were also associated with Kyoto Encyclopedia of Genes and Genomes (KEGG) pathways including hypertrophic cardiomyopathy, calcium signaling pathway, oxytocin signaling pathway, and drug metabolism (Figure 3a,b; Table S3). Additionally, the downregulated genes were enriched in molecular function (MF) annotations related to channel and transporter activity, muscle protein binding, and protein phosphatase regulator activity (Figure 4a,b; Table S4).

HFD upregulated genes in BAT of HFD-fed mice were significantly associated with BP annotations related to immune responses, such as leukocyte and macrophage chemotaxis, T cell-mediated immunity, and lymphocyte proliferation (Figure 2a,c; Table S5). These genes were also significantly associated with KEGG pathways such as natural killer cell mediated cytotoxicity, toll-like receptor signaling pathway, and allograft rejection (Figure 3a,c; Table S6). Additionally, MF annotations related to chemokine activity and SH3/SH2 adaptor activity were significantly increased in BAT of HFD-fed mice (Figure 4a,c; Table S7). A group of chemokine-encoding genes were significantly upregulated in BAT of HFD-fed mice, including *Ccl4*, *Ccl5*, *Cx3cl1*, *Cxcl10*, and *Xcl1*. Additionally, the expression of *Tnf* was increased in BAT of HFD-fed obese mice (log2 fold change = 2.1229; *P*_adj_ = 0.0274).

### 2.3. Transcript Levels of BAT Morphogenic Genes, Mitochondrial Genes, and Thermogenic Genes

HFD feeding significantly upregulated morphogenic genes for developmental regulation such as *Bmp8b* (log2 fold change = 2.5433; *P*_adj_ = 0.0014; Table S8), and downregulated *Fgf10* (log2 fold change = 1.8028; *P*_adj_ = 0.0372) and *Fgf2* (log2 fold change = 1.6354; *P*_adj_ = 0.0805) in BAT. HFD feeding downregulated the Cox6a2 gene encoding cytochrome c oxidase (COX) that functions in electron transfer in mitochondrial respiration [40] (log2 fold change = 3.8812; *P*_adj_ = 1.3019 × 10^−9^; Table S8). Additionally, HFD feeding upregulated two genes that code for fatty acid-binding proteins for trafficking fatty acids to mitochondria and peroxisomes for oxidation or to lipid droplets for storage [41], *Fabp3* (log2 fold change = 2.5858; *P*_adj_ = 0.0005) and *Fabp7* (log2 fold change = 2.2040; *P*_adj_ = 0.0034). These data suggested BAT mitochondrial dysfunction following HFD-induced obesity.

High abundance of a few key thermogenic genes were expressed in BAT samples and were upregulated by HFD feeding, including *Ucp1* (log2 fold change = 1.9507; *P*_adj_ = 0.1189), *Ppara* (log2 fold change = 1.8855; *P*_adj_ = 0.0592), *Prdm16* (log2 fold change = 1.1483; *P*_adj_ = 0.1981), *Ppargc1a* (log2 fold change = 1.1229; *P*_adj_ = 0.4928), but the differential expression was not significant in the BAT between the two dietary groups. Additionally, none of the regulatory genes related to thermogenesis [42] was significantly changed in BAT by HFD feeding, including *Ebf2*, *Ehmt1*, *Pparg*, and *Tle3*. Therefore, our results indicated that expression of the aforementioned BAT thermogenic genes was not significantly affected by HFD-induced obesity.

### 2.4. Validation of RNA-Seq Data Using Reverse Transcription-Quantitative PCR (RT-qPCR)

**Figure 5:**
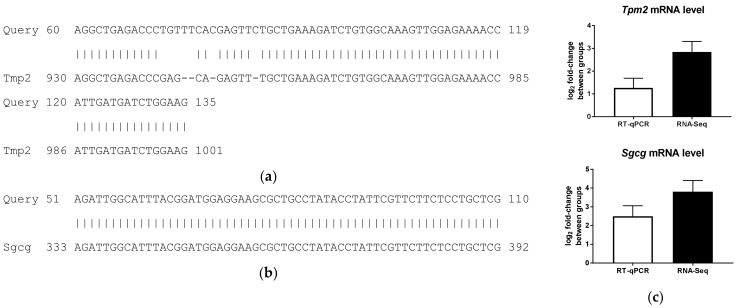
Validation of RNA sequencing (RNA-Seq)-based expression of tropomyosin 2 (*Tmp2*) and sarcoglycan gamma (*Sgcg*) genes using reverse transcription-quantitative PCR (RT-qPCR). (**a**) PCR product *Tmp2* sequenced using Sanger sequencing and mapped to NCBI Blast revealed 91% match with 69/76 match and 4/76 gaps; (**b**) PCR product *Sgcg* sequenced using Sanger sequencing and mapped to NCBI Blast revealed 100% match with 60/60 match and no gap; (**c**) RNA-Seq- and RT-qPCR-determined log_2_ fold-changes of *Tpm2* and *Sgcg* genes.

The novel finding of the current study is reduced expression of genes involved in muscle structure and muscle system process in the BAT of HFD-fed mice, including genes encoding tropomyosin β (*Tpm2*) and sarcoglycan gamma (*Sgcg*). To our knowledge, none of the previous microarray studies had identified differential expression of genes related to muscle structure or muscle system process in the BAT of HFD-induced obese mice [27,28,29,30]. Glyceraldehyde-3-phosphate dehydrogenase (*Gapdh*) was not differentially expressed in BAT of LFD and HFD mice, supported by the RNA-Seq data (log2 fold change = 0.3866; *P*_adj_ = 0.9975), and, thus, was used as a control. Two downregulated genes by HFD feeding, *Tpm2* and *Sgcg*, identified by RNA-Seq were validated using RT-qPCR. In order to confirm the specificity of the PCR primers (Table 2), amplified products were sequenced using conventional Sanger sequencing, and mapped to NCBI Blast for *Tpm2* and *Sgcg*. Specifically, the mapping identities were 91% for *Tpm2* (Figure 5a) and 100% for *Sgcg* (Figure 5b), indicating specific RT-qPCR primers.

*Tpm2* expression in BAT of HFD-fed mice was reduced compared to BAT of LFD-fed mice using RNA-Seq (log2 fold change = 2.8394 ± 0.4623; *P*_adj_ = 4.4779 × 10^−7^) and PCR result indicated significantly less expression in HFD-fed mice than LFD-fed mice (log2 fold change = 1.2604 ± 0.4307; *P* < 0.05). *Sgcg* expression of BAT of HFD-fed was downregulated compared to LFD-fed mice using RNA-Seq (log2 fold change = 3.8037 ± 0.6006; *P*_adj_ = 1.7411 × 10^−7^), and PCR validated the downregulation following HFD feeding (log2 fold change = 2.4959 ± 0.5657; *P* < 0.05). Additionally, the log_2_ fold-changes of mRNA levels of *Tpm2* and *Sgcg* obtained using these two methods, RT-qPCR and RNA-Seq, were not significantly different. As a result, RNA-Seq and PCR-determined expression levels were in good agreement (Figure 5c).

## 3. Discussion

Obesity, characterized by increases in body and fat mass, has become a global health problem. It is widely accepted that HFD consumption is one of the major causes for this increased worldwide prevalence of obesity. Obesity leads to the development of many other metabolic diseases such as cardiovascular diseases, type 2 diabetes, and low-grade chronic inflammation. Therefore, the impact of diet-induced obesity on adipose tissues is of great interest. WAT serves as the main fat storage depot. BAT, a mitochondria-rich type of adipose tissue with high thermogenic capacity, is specialized for heat generation and consumption of stored energy [4]. Recent studies have conclusively demonstrated that adult humans possess metabolically active BAT whose activity is increased by cold exposure [7,8,9,43]. Given the ability of BAT to burn calories, research attention focused on BAT has been growing in the biomedical community because of its therapeutic potential for accelerating weight loss in obese individuals [44,45].

In this study, mice fed a HFD for four weeks developed diet-induced obesity with greater body mass and fat mass than the mice fed a LFD. HFD-induced obesity in C57BL/6J mice has been reported by many groups, including our group [46,47,48], which is partially caused by increased caloric intake and reduced energy expenditure [47]. Fewer studies, however, have focused on the molecular modification of BAT than WAT during the development of HFD-induced obesity. We used RNA-Seq to perform transcriptome analysis of BAT of lean mice fed a LFD and obese mice fed a HFD, identified their differentially expressed genes, and evaluated associated pathways and potential mechanisms underlying BAT metabolic function in a diet-induced obesity model. The depth of transcriptomic detail provided by our study identified downregulation of genes involved in ion transport, mitochondrial biogenesis, and muscle development and process; and concurrent upregulation of genes involved in immune responses, fatty acid uptake, and BAT differentiation by HFD-induced obesity.

Some, but not all, findings from the current study, are consistent with the findings from previous studies that used microarray to identify differentially expressed genes in the BAT of HFD-induced obese mice [27,28,29,30]. An earlier study by Fitzgibbons et al. compared global differences in the full genome set of gene expression in the BAT from male C57BL6/J mice that were fed with a normal diet and a 45% HFD for 13 or 20 weeks [30]. Three biological replicates per dietary group were analyzed using microarray. Pathway analysis was not available in this study. The major finding was that BAT of HFD group had lower expression of immune cell-enriched genes, indicating resistance of BAT to HFD-induced inflammation [30], which does not agree with findings from the current study. Fitzgibbons et al. also reported that the BAT of two dietary groups did not show significantly different expression in the majority of genes that are highly expressed in BAT such as *Ucp1* [30], which is consistent with the current finding. Interestingly, BAT had enriched expressions of the genes related to skeletal muscle differentiation and function [30], which was also seen in the current study. Svahn et al. [27] analyzed genes of BAT of C57BL/6J mice that were fed with a 60% HFD or a 10%LFD for 8 weeks using four biological replicates per dietary group for microarray analysis. The “piano” package was used to perform gene-set analysis focusing on the GO terms under the “immune system process”. Interestingly, the immune system GO-term was not altered in BAT of the HFD-fed mice compared to the mice fed with the LFD, which is consistent with the findings reported by Fitzgibbons et al. [30], but not with the findings from the current study. Such discrepancy could be due to different time courses of HFD feeding. Kim et al. analyzed genes in the BAT of C57BL/6J mice that were fed with a HFD with unspecified fat component and a normal diet for various durations up to 24 weeks, using 3 biological replicates per dietary group at each time point for microarray analysis [29]. The functional annotation tool DAVID (http://david.abcc.ncifcrf.gov/; [49]) GO mining and pathway analysis was used, and the distribution of curated gene sets obtained from the molecular signature database were compared using gene set enrichment analysis (MSigDB v4.0, GSEA software, Broad Institute, Cambridge, MA, USA) [29]. The major finding is that expressions of genes related to immune response and lipid metabolism change throughout HFD-induced obesity development [29]. Particularly, upregulation of immune-response genes was seen in the BAT at 2 and 4 weeks, but not between 8- and 20-week time points, and “reappeared” at 24 weeks after HFD feeding [29]. This agrees with the finding from this study showing upregulation of immune response genes in the BAT 4 weeks after HFD feeding, and also agrees with findings from two previous studies that failed to show change in immune response genes in the BAT after HFD feeding for 8–20 weeks [27,30].

The novel finding of the current study is significantly reduced expression of a few genes involved in muscle structure and muscle system process in the BAT by HFD feeding, including genes encoding tropomyosin β (*Tpm2*) and sarcoglycan gamma (*Sgcg*). None of the previous microarray studies had identified differential expression of genes related to muscle structure and process in the BAT of HFD-induced obese mice [27,28,29,30]. Brown adipocytes and myocytes develop from a common adipocyocyte precursor [50]. *Tpm2* and *Sgcg* are known as muscle structure-related BAT genes [51]. Both tropomyosin and sarcoglycan are components of cytoskeleton in preadipocytes [52] and in white and brown adipocytes [53].

All the previous studies that used microarray to examine differential expression of genes in BAT of HFD-induced obese mice used three or four biological replicates [27,28,29,30]. The advantage for using small sample size for statistical testing is that the uncovered significant difference actually exists, because larger samples usually increase the chance of significance and often generate overwhelmingly long lists of differentially expressed genes that might not be biologically meaningful [54]. In the current study, RNA expression analysis was performed at the gene level, and expression data were normalized with DESeq2. DESeq2 is an advanced methodology that analyzes comparative RNA-seq data using shrinkage estimators for fold change, standard error, and dispersion, and provides quality assessment and clustering of overdispersed data [54]. Besides accounting for non-normality and dependence of variance of RNA-seq data, DESeq2 is highly sensitive and precise for using small sample sizes, often as few as two or three replicates per experimental condition, in typical high-throughput sequencing studies [23,55,56], including the first hit [57] of the current PubMed search (Table S1).

### 3.1. Functional Significance of Differentially Expressed Morphogenic Genes

High expression levels of thermogenic gene *Ucp1* and transcriptional regulatory genes related to thermogenesis, such as *Prdm16*, *Pparg*, *Ppargc1a*, *Ebf2*, *Ehmt1*, and *Tle3*, were not significantly changed in BAT by HFD feeding. The mice were housed either under “standard condition” (current study and [27]), or at unspecified temperatures [29,30] but most likely would be at an ambient temperature of around 22 °C, rather than at thermoneutral condition of 30 °C. Although BAT dysfunction and downregulation of thermogenic genes are hypothetically expected, expressions of *Ucp1* and other thermogenic genes in the BAT were not different between dietary groups in any of the previous studies [27,29,30] or the current study. BAT thermogenic genes are upregulated to activate thermogenesis and maintain body temperature when mice are housed at an ambient temperature [58], which is counterregulatory to the potential downregulatory effects of HFD on BAT thermogenic genes. Another possible explanation is that BAT maintains its function in thermogenesis during HFD-induced obesity, as one study reported that BAT of the mice fed a 60% HFD for 12 weeks and housed at 30 °C had normal BAT morphology compared with LFD-fed mice upon histological examination [28]. A brown adipocyte signature gene related to mitochondrial respiratory chain Cox6a2 [40] was downregulated in BAT of HFD-fed mice relative to LFD-fed mice, suggesting that BAT of HFD has suppressed mitochondrial respiratory function and reduced fatty acid oxidation, potentially leading to suppressed thermogenesis and reduced energy expenditure. Bone morphogenetic protein 8B (BMP8B) not only plays a role in differentiation but also signals to mature brown adipocytes and regulates BAT thermogenesis [59]. Morphogenic gene *Bmp8b* involved in cell fate regulation during development [19] were upregulated in BAT of HFD-fed mice relative to LFD-fed mice, possibly to compensate downregulated respiratory function to maintain thermogenesis.

### 3.2. Functional Significance of Differentially Expressed Genes for Fatty Acid Uptake

BAT takes up free fatty acids from triglyceride-rich lipoproteins, which is activated by cold exposure in mice, resulting in accelerated plasma triglyceride clearance [13]. Two fatty acid binding protein genes, *Fabp3* and *Fabp7*, were upregulated in BAT by HFD feeding, indicating increased fatty acid uptake into BAT [60]. *Fabp3* and *Fabp7* code fatty acid-binding proteins (FABPs) that act as fatty acid chaperones for trafficking fatty acid to mitochondria for thermogenesis and to lipid droplets for storage [41]. FABP3 is induced by acute cold exposure in BAT of rats [61,62], which suggests that FABPs may regulate the utilization of fatty acids, via fatty acid trafficking and oxidation, for thermogenesis in BAT. Additionally, BAT of HFD-induced obese mice may take on an additional role in fat storage that is typical to WAT. This is consistent with previous reports showing that the role of BAT in HFD-induced obesity could be changed to mirror WAT as a storage depot of excess energy which potentially contributes to BAT whitening [63].

The oxytocin signaling pathway was downregulated by HFD feeding. Oxytocin acts on its receptor, a G protein-coupled receptor coupled to the G protein with Gαq, leading to activation of phospholipase C and generation of inositol triphosphate to trigger Ca^2+^ release from endoplasmic reticulum, and increased cytosolic Ca^2+^ concentration, to initiate muscle contraction or use Ca^2+^ as a second messenger. It is established that the oxytocin signaling acts both centrally and peripherally [64] to reduce body adiposity via increasing energy expenditure [65] and to inhibit preadipocyte differentiation and adipogenesis [66].

### 3.3. Functional Significance of Differentially Expressed Genes for Ion Transport

The expression of genes associated with ion transport and muscle development were found negatively correlated with diet-induced obesity in this study. BAT cells maintain ion concentration differences across their membranes. BAT cells depolarize using multiple types of ion flux, including release of intracellular Ca^2+^ and transmembrane flux of Cl^−^, Na^+^, and K^+^ currents [4]. HFD feeding reduced expressions of genes coding for channels and regulators for Ca^2+^ (*Atp2a1*, *Cacna1s*, *Cacna2d1*, *Cacnb1*, *Cacng1*, *Cacng6*, *Hrc*, *Jph2*, *Jsrp1*, *Ryr1*, *Slc8a3, Trdn*), Cl^−^ (*Clcn1*), K^+^ (*Kcna7*, *Kcnc1*, *Kcnc4*, *Kcnj12*), and Na^+^ (*Scn1b*, *Scn4a*, *Slc8a3*) in BAT. Additionally, while HFD feeding significantly decreased *Ryr1* expression, it enhanced *Ryr2* expression. Intracellular Ca^2+^ is an important second messenger for signal transduction and is essential for cellular processes such as excitation-contraction coupling. *Atp2a1* codes endoplasmic reticulum Ca^2+^-ATPase 1, which transports Ca^2+^ into the endoplasmic reticulum and controls the level of Ca^2+^ inside cells. Endoplasmic reticulum Ca^2+^-ATPase 1 expression represents a source of heat production contributing to BAT thermogenic function [67]. The Ca^2+^-permeable intracellular channels ryanodine receptor 1 (RyR1) and 2 (Ryr2) regulate Ca^2+^ release from the endoplasmic reticulum into the cytosol and Ca^2+^-cycling required excitation-contraction coupling [68,69]. RyR1 and RyR2 also have been implicated in the regulation of thermogenesis in mice [70,71]. Furthermore, *Cacna2d1* encoded for a voltage-dependent Ca^2+^ channel has been reported to be associated with body fat level [72].

### 3.4. Functional Significance of Differentially Expressed Genes for Inflammation

Obesity is associated with chronic low-grade inflammation. Excess delivery of nutrients when subjects are fed with a HFD increases in lipid storage in adipose tissues, followed by increased infiltration and activity of immune cells such as macrophages and lymphocytes, as well as increased production of inflammatory chemokines and cytokines, ultimately contributing to the progression of metabolic dysfunction [73,74] and negatively impact on human health through mechanisms such as promoting insulin resistance [75] and antherogenesis [76].

In this study, expression levels of the T lymphocyte and natural killer cell marker gene *Cd69*, chemokine-encoded gene *Ccl5* [77,78], chemoattractant-encoded gene *Ccl4* [79], cytokine interferon gamma inducible chemokine-encoded genes *Cxcl9* and *Cxcl10* [80], and tumor necrosis factor α (TNFα)-encoded gene *Tnf* were upregulated in the BAT of HFD-fed obese mice. The functional annotation analyses revealed that genes associated with immune responses, chemotaxis of leukocytes and macrophages, T cell-mediated immunity, and lymphocyte proliferation were upregulated, indicating that BAT of obese mice exhibited immune cell activation and severe immune responses, leading to chronic low-grade inflammation in BAT. These findings help unravel the molecular mechanism regulating obesity-induced adipose tissue inflammation and related metabolic dysfunction such as insulin resistance.

The current finding showing inflammation induced by high-fat feeding in BAT is not surprising, as many previous reports have indicated that increased infiltration of macrophages and T cells into BAT [18,81,82] could be detected in mouse models. Our finding is consistent with a previous report showing upregulation of immune response genes in BAT following four weeks of HFD feeding using microarray [29]. Some previous studies using microarray, however, reported no significant change in immune cell-expressed genes or enriched GO-term associated with immune system following 8 weeks [27] or up to 20 weeks of HFD feeding [30]. As mentioned above, Such discrepancy could be due to different time courses of HFD feeding, as immune-response genes have been reported to be upregulated in the BAT at early (<4 weeks) and late (24 weeks) stages of HFD feeding, but not changed between 8 and 20 weeks of HFD feeding [29]. Future studies attempting to understand underlying mechanisms are warranted.

The RNA-Seq method was applied in this study with a broader dynamic range and higher sensitivity than the microarray technique, providing us with a comprehensive profile of obesity-induced inflammatory changes in gene expression of BAT. With more than 80 million obese adults in the U.S., and a national obesity rate of 35% [1], the presence of functionally active BAT in adult humans has received considerable attention due to its unique ability to burn calories. HFD feeding leads to obesity and increases risk factors for many diseases. The excitement associated with BAT as a potential means to combat obesity requires us to further investigate the molecular mechanisms for BAT dysfunction affected by HFD-induced obesity.

## 4. Materials and Methods

### 4.1. Animals and Diets

Six-week old male C57BL/6J mice (Stock Number 000664, Jackson Laboratory, Bar Harbor, ME, USA) were single-housed with a 12 h light/12 h dark cycle and 22 ± 1 °C room temperature. Mice were grouped into two groups (n = 5/group) with matched average body weights and body composition. One group of mice was fed a LFD (3.35 kcal/g; 5% calories from fat; laboratory rodent diet, 5001, LabDiet, St. Louis, MO, USA) and the other group fed a HFD (4.728 kcal/g; 45% calories from fat; D12451, Research Diets, New Brunswick, NJ, USA) for four weeks. All animal experiments were approved by the Institutional Animal Care and Use Committee, Miami University Ohio on 24 May, 2010 (protocol #801), and were conducted according to the guidelines of U.S. National Institutes of Health.

### 4.2. Body Mass and Body Composition

Body mass and body composition were measured before and at the end of HFD feeding. Body composition was assessed using an EchoMRI body composition analyzer (EchoMedical Systems, Houston, TX, USA) which measures the whole-body fat and lean mass.

### 4.3. RNA Extraction and Quality Control

Mice were euthanized at the end of 4-week feeding. Three of five mice from each dietary group with greatest, medium, and least fat mass were used for RNA-Seq in the current study. In order to be consistent, right side of BAT at the interscapular region was collected, weighed, flash frozen, and stored at −80 °C. Total RNA was isolated for sequencing using E.Z.N.A^®^ Total RNA kit II (Omega Bio-tek, Norcross, GA, USA) according to the manufacturer’s instructions. Briefly, ~20 mg BAT were homogenized in 1 mL of RNA-solv reagent using bullet blender^®^ tissue homogenizer (Next Advance Inc., Averill Park, NY, USA). The homogenized tissues were then processed with chloroform addition and phase separation. The aqueous phase was transferred to a new sterile 1.5 mL tube and mixed with equal volume of 70% ethanol. The mixtures were loaded to the Hibind^®^ RNA spin column and processed according to the manufacturer’s instruction. Total RNA was eluted in 30 μL nuclease-free water and stored in −80 °C. The concentration and purity of extracted RNA samples were checked using a NanoDrop^TM^ spectrophotometer (Thermo Fisher Scientific Inc., Waltham, MA, USA), then analyzed using an Agilent RNA 6000 pico kit with an Agilent’s 2100 Bioanalyzer (Agilent Technologies Inc., Waldbronn, Germany). High quality of RNA samples with an RNA integrity number (RIN) above 6.5 were used for analysis.

### 4.4. RNA-Seq

Ribosomal RNA was removed by using TruSeq Standard Total RNA Library Prep Kit with Ribo-Zero Gold (Illumina). The libraries were sequenced as single-end 75 bp on Illumina NextSeq500 sequencing system at Cofactor Genomics Inc. (St. Louis, MO, USA). Data retrieved from single-end sequencing is appropriate for identifying differential gene expressions between HFD- and LFD-mice, as mouse is a model organism with reference genome available for mapping; whereas pair-end sequencing improves read alignment and helps to resolve chromosomal rearrangements, thus is relatively suitable for de novo analysis of a non-model organism that lacks a reference genome. Approximately 30 million of reads were retrieved from each sample (Table 1).

### 4.5. Sequence Read Processing and Mapping

After removal of Truseq3-SE adapter sequence (AGATCGGAAGAGCGTCGTGTAGGGAAAGAGTGTA) using Trimmomatic (version 0.36) [83], single-end sequencing reads were aligned to the mouse genome (ENSEMBL 84 release, GRCm38.p4) using RSEM (version 1.3.0) [84]. Differential gene expression analysis was performed by DESeq2 using the estimated gene-level counts from RSEM [54]. RNA expression analysis was performed at the gene level, and expression data were normalized with DESeq2.

All the genes produced by the DESeq2 differential analysis were ranked by the log2 fold change values (Table S8), because some genes with small fold changes could be statistically significant but may not be promising candidates for further investigation. The sequence data were deposited at Gene Expression Omnibus (GEO), at https://www.ncbi.nlm.nih.gov/geo/info/seq.html, a publicly available database. The accession number is GSE112740.

### 4.6. Transcriptome and Functional Analysis

Transcriptome analysis identified distinct gene transcripts that mapped to mouse genome (ENSEMBL 84 release, GRCm38.p4) and displayed significantly differential expression in BAT between LFD- and HFD-fed mice using adjusted *P* value (*P*_adj_) < 0.01 and a |log2 fold change| > 1.5. Next we investigated biological characteristics associated with these genes using ClueGO (http://apps.cytoscape.org/apps/cluego), a functional classification tool that uses precompiled annotation files including GO enrichment analysis [85]. GO annotates genes to BP, KEGG, and MF terms in a hierarchically structured way [86] and assigns genes to functional pathways [87]. Terms with a *P* value < 0.05 were considered significant.

### 4.7. Reverse Transcription-Quantitative PCR (RT-qPCR)

Some differentially expressed genes revealed by RNA-Seq were validated with RT-qPCR using the samples that were sequenced. Briefly, 200 ng RNA was reverse transcribed using iScript^TM^ cDNA synthesis kit (Bio-Rad Laboratories Inc., Hercules, CA, USA) according to manufacturer’s instructions. cDNA was purified by Wizard^®^ SV Gel and PCR Clean-up system (Promega, Madison, WI, USA). The synthesized cDNA was then amplified by specific primers for a housekeeping gene *Gapdh* used as the internal control as it was not differentially expressed in BAT of LFD and HFD mice (see Results), tropomyosin 2 (*Tpm2*), and sarcoglycan gamma (*Sgcg*) (Table 2), using the following conditions: 95 °C, 2 min; 35 cycles of amplification at 95 °C, for 30 s; 55 °C, for 30 s; 72 °C, for 30 s; then polish at 72 °C for 10 min. In order to confirm the specificity of the primers, amplified products were separated on a 2% agarose gel, and the bands were purified and sequenced using conventional Sanger sequencing. Amplicon from each gene were purified from the agarose gel and sequenced with the BigDye™ Cycle Sequencing kit (Applied Biosystems) according to manufacturer’s instructions, and run on an ABI Prism 3730xl automatic sequencer (Applied Biosystems).

RT-qPCR was performed on Bio-Rad CFX96TM Real-Time PCR Detection System using iQ^TM^ SYBR^®^ Green Supermix (Bio-Rad, Hercules, CA, USA), 40 ng of cDNA as templates for each reaction, and was run in triplicates. The PCR program was set at 95 °C for 2 min, 40 cycles of amplification at 95 °C for 30 s, and annealing at 55 °C for 30 s. *Tpm2* and *Sgcg* expression was normalized to housekeeping gene *Gapdh*. Relative mRNA expression level of each gene was calculated using the ΔCt (2^−ΔCt^) method [88], and presented using LFD group as 100%.

### 4.8. Statistical Analysis

The differentially expressed genes between mice fed with LFD and HFD were analyzed using DESeq2 package in R statistical language, with a |log2 fold change| > 1.5 and *P*_adj_ < 0.01 considered to be statistically significant. Body mass, BAT mass, body composition, gene expression from quantitative PCR data were presented as mean ± SEM, and LFD and HFD groups were compared using unpaired Student’s *t*-test (GraphPad Prism 7; La Jolla, CA, USA). *P* values < 0.05 was considered statistically significant.

## 5. Conclusions

Collectively, the effects of HFD-induced obesity on the BAT transcriptome include downregulation of genes related to muscle development, muscle system process, ion transport, and neurotransmitter secretion, and upregulation of genes related to immune responses, fatty acid uptake, and BAT differentiation. Regulatory genes related to thermogenesis were not significantly changed in BAT of HFD-induced obese mice, suggesting that despite an increased inflammation in BAT, BAT maintained its thermogenic function following four weeks of HFD feeding. Findings from this study provide mechanistic insight into the role of BAT during the development of diet-induced obesity, and the importance of selecting BAT as a target for potential therapeutic interventions of obesity and related metabolic disorders.

## Figures and Tables

**Table 1:** Summary of RNA sequencing (RNA-Seq) data with adapter removal and quality trimming.

Sample	Total Raw Reads	High-Quality Reads	Low-Quality Reads	High-Quality-Adapter Reads	Clean %
HFD rep1	28,474,259	28,185,294	288,965	28,182,180	98.97%
HFD rep2	28,734,314	28,479,650	254,664	28,476,939	99.10%
HFD rep3	29,120,330	28,827,315	293,015	28,824,200	98.98%
LFD rep1	27,811,961	27,521,844	290,117	27,515,579	98.93%
LFD rep2	31,346,988	31,000,262	346,726	30,996,159	98.88%
LFD rep3	29,392,169	28,998,251	393,918	28,991,538	98.64%

**Table 2:** Reverse transcription-quantitative PCR primer sequences. Glyceraldehyde-3-phosphate dehydrogenase (*Gapdh*), tropomyosin 2 (*Tpm2*), sarcoglycan gamma (*Sgcg*).

Genes	GenBank Accession Number	Forward and Reverse Primer Sequences
*Gapdh*	NC_000072	F: 5′-GCGACTTCAACAGCAACTC-3′ R: 5′-GCCTCTCTTGCTCAGTGTCC-3′
*Tpm2*	NC_000070	F: 5′-GGCAGGAAACTGAGGGGTAG-3′ R: 5′-GCAGGGGAGTCCTTTTTACCT-3′
*Sgcg*	NC_000080	F: 5′-TCACCGAGGGCACTCACATA-3′ R: 5′-CGAGCAGGAGAAGAACGAATAGG-3′

## Supplementary Materials

Supplementary materials can be found at http://www.mdpi.com/1422-0067/19/4/1095/s1.

## Abbreviations

BP	Biological processes
BAT	Brown adipose tissue
GO	Gene Ontology
HFD	High-fat diet
KEGG	Kyoto Encyclopedia of Genes and Genomes
LFD	Low-fat diet
MF	Molecular function
