# Supplementary material for: Global Transcriptome Analysis of Brown Adipose Tissue of Diet-Induced Obese Mice
Source: Int J Mol Sci. 2018 Apr 6;19(4):1095. doi: 10.3390/ijms19041095 (PMC5979511; doi:10.3390/ijms19041095)
Supplement: Supplementary file 1 [file ijms-19-01095-s001.zip › Table S1.pdf]

PubMed Home | More Resources | Help

# PubMed Advanced Search Builder

Tutorial

Use the builder below to create your search

Edit

Clear

## Builder

All Fields ▼

AND ▼ All Fields ▼

Show index list

Show index list

Search or Add to history

## History

Download history Clear history

| Search | Add to builder | Query | Items found | Time |
|---|---|---|---|---|
| #3 | Add | Search **(((brown adipose tissue) AND high fat diet)) AND ((((microarray) OR RNA sequencing) OR RNA seq) OR sequencing)** | 26 | 11:24:43 |
| #2 | Add | Search **(((microarray) OR RNA sequencing) OR RNA seq) OR sequencing** | 439780 | 11:24:27 |
| #1 | Add | Search **(brown adipose tissue) AND high fat diet** | 1069 | 11:23:53 |

PubMed ▼ (((brown adipose tissue) AND high fat diet)) AND ((((microarray) OR RN

**Format**: Summary **Sort by**: Most Recent **Per page**: 50

## Search results

**Items: 26**

1. Cdkn2a deficiency promotes **adipose tissue** browning.
Rabhi N, Hannou SA, Gromada X, Salas E, Yao X, Oger F, Carney C, Lopez-Mejia IC, Durand E, Rabearivelo I, Bonnefond A, Caron E, Fajas L, Dani C, Froguel P, Annicotte JS.
Mol Metab. 2017 Dec 1. pii: S2212-8778(17)30935-3. doi: 10.1016/j.molmet.2017.11.012. [Epub ahead of print]
PMID: 29237539 **Free Article**

2. Transcriptomic responses of the liver and **adipose** tissues to altered carbohydrate-**fat** ratio in **diet**: an isoenergetic study in young rats.
Tanaka M, Yasuoka A, Shimizu M, Saito Y, Kumakura K, Asakura T, Nagai T.
Genes Nutr. 2017 Apr 8;12:10. doi: 10.1186/s12263-017-0558-2. eCollection 2017.
PMID: 28405243 **Free PMC Article**

3. 17β-Estradiol suppresses visceral adipogenesis and activates **brown adipose tissue**-specific gene expression.
Al-Qahtani SM, Bryzgalova G, Valladolid-Acebes I, Korach-André M, Dahlman-Wright K, Efendić S, Berggren PO, Portwood N.
Horm Mol Biol Clin Investig. 2017 Jan 1;29(1):13-26. doi: 10.1515/hmbci-2016-0031.
PMID: 27831918

4. Pharmacologic activation of estrogen receptor β increases mitochondrial function, energy expenditure, and **brown adipose tissue**.
Ponnusamy S, Tran QT, Harvey I, Smallwood HS, Thiyagarajan T, Banerjee S, Johnson DL, Dalton JT, Sullivan RD, Miller DD, Bridges D, Narayanan R.
FASEB J. 2017 Jan;31(1):266-281. doi: 10.1096/fj.201600787RR. Epub 2016 Oct 12.
PMID: 27733447 **Free PMC Article**

5. Six **Tissue** Transcriptomics Reveals Specific Immune Suppression in Spleen by Dietary Polyunsaturated Fatty Acids.
Svahn SL, Väremo L, Gabrielsson BG, Peris E, Nookaew I, Grahnemo L, Sandberg AS, Wernstedt Asterholm I, Jansson JO, Nielsen J, Johansson ME.
PLoS One. 2016 May 11;11(5):e0155099. doi: 10.1371/journal.pone.0155099. eCollection 2016.
PMID: 27166587 **Free PMC Article**

6. Fucoidan alleviates **high-fat diet**-induced dyslipidemia and atherosclerosis in ApoE(shl) mice deficient in apolipoprotein E expression.
Yokota T, Nomura K, Nagashima M, Kamimura N.
J Nutr Biochem. 2016 Jun;32:46-54. doi: 10.1016/j.jnutbio.2016.01.011. Epub 2016 Mar 8.
PMID: 27142736

7. Loss of **Adipose** Fatty Acid Oxidation Does Not Potentiate Obesity at Thermoneutrality.
Lee J, Choi J, Aja S, Scafidi S, Wolfgang MJ.
Cell Rep. 2016 Feb 16;14(6):1308-1316. doi: 10.1016/j.celrep.2016.01.029. Epub 2016 Feb 4.
PMID: 26854223 **Free PMC Article**

8. Transcriptome profiling of white **adipose tissue** in a mouse model for 15q duplication syndrome.
Liu X, Tamada K, Kishimoto R, Okubo H, Ise S, Ohta H, Ruf S, Nakatani J, Kohno N, Spitz F, Takumi T.
Genom Data. 2015 Jul 10;5:394-6. doi: 10.1016/j.gdata.2015.06.035. eCollection 2015 Sep.
PMID: 26484295 **Free PMC Article**

9. Gene expression profiles reveal effect of a **high-fat diet** on the development of white and **brown adipose** tissues.
Kim HS, Ryoo ZY, Choi SU, Lee S.
Gene. 2015 Jul 1;565(1):15-21. doi: 10.1016/j.gene.2015.03.077. Epub 2015 Apr 17.
PMID: 25895476

10. A gain-of-function mutation in adenylate cyclase 3 protects mice from **diet**-induced obesity.
Pitman JL, Wheeler MC, Lloyd DJ, Walker JR, Glynne RJ, Gekakis N.
PLoS One. 2014 Oct 16;9(10):e110226. doi: 10.1371/journal.pone.0110226. eCollection 2014.
PMID: 25329148 **Free PMC Article**

11. MitoNEET-mediated effects on browning of white **adipose tissue**.
Kusminski CM, Park J, Scherer PE.
Nat Commun. 2014 May 28;5:3962. doi: 10.1038/ncomms4962.
PMID: 24865177 **Free PMC Article**

12. **Adipose**-specific knockout of SEIPIN/BSCL2 results in progressive lipodystrophy.
Liu L, Jiang Q, Wang X, Zhang Y, Lin RC, Lam SM, Shui G, Zhou L, Li P, Wang Y, Cui X, Gao M, Zhang L, Lv Y, Xu G, Liu G, Zhao D, Yang H.
Diabetes. 2014 Jul;63(7):2320-31. doi: 10.2337/db13-0729. Epub 2014 Mar 12.
PMID: 24622797 **Free Article**

13. Hkat, a novel nutritionally regulated transmembrane protein in **adipose** tissues.
Zhang R.
Sci Rep. 2012;2:825. doi: 10.1038/srep00825. Epub 2012 Nov 14.
PMID: 23152936 **Free PMC Article**

14. Nrac, a novel nutritionally-regulated **adipose** and cardiac-enriched gene.
Zhang R, Yao F, Gao F, Abou-Samra AB.
PLoS One. 2012;7(9):e46254. doi: 10.1371/journal.pone.0046254. Epub 2012 Sep 27.
PMID: 23029450 **Free PMC Article**

15. Extrinsic and intrinsic regulation of DOR/TP53INP2 expression in mice: effects of dietary **fat** content, **tissue** type and sex in **adipose** and muscle tissues.
Fromm-Dornieden C, Lytovchenko O, von der Heyde S, Behnke N, Hogl S, Berghoff J, Köpper F, Opitz L, Renne U, Hoeflich A, Beissbarth T, Brenig B, Baumgartner BG.
Nutr Metab (Lond). 2012 Sep 21;9(1):86. doi: 10.1186/1743-7075-9-86.
PMID: 22995226 **Free PMC Article**

16. Inhibition of myostatin protects against **diet**-induced obesity by enhancing fatty acid oxidation and promoting a **brown adipose** phenotype in mice.
Zhang C, McFarlane C, Lokireddy S, Masuda S, Ge X, Gluckman PD, Sharma M, Kambadur R.
Diabetologia. 2012 Jan;55(1):183-93. doi: 10.1007/s00125-011-2304-4. Epub 2011 Sep 17. Erratum in: Diabetologia. 2015 Mar;58(3):643.
PMID: 21927895

17. Disruption of hypoxia-inducible factor 1 in adipocytes improves insulin sensitivity and decreases adiposity in **high-fat diet**-fed mice.
Jiang C, Qu A, Matsubara T, Chanturiya T, Jou W, Gavrilova O, Shah YM, Gonzalez FJ.
Diabetes. 2011 Oct;60(10):2484-95. doi: 10.2337/db11-0174. Epub 2011 Aug 26.
PMID: 21873554 **Free PMC Article**

18. Similarity of mouse perivascular and **brown adipose** tissues and their resistance to **diet**-induced inflammation.
Fitzgibbons TP, Kogan S, Aouadi M, Hendricks GM, Straubhaar J, Czech MP.
Am J Physiol Heart Circ Physiol. 2011 Oct;301(4):H1425-37. doi: 10.1152/ajpheart.00376.2011. Epub 2011 Jul 15.
PMID: 21765057 **Free PMC Article**

19. Gene expression profiling of **adipose** tissues in obesity susceptible and resistant rats under a **high fat diet**.
Joo JI, Yun JW.
Cell Physiol Biochem. 2011;27(3-4):327-40. doi: 10.1159/000327959. Epub 2011 Apr 1.
PMID: 21471722 **Free Article**

20. Pharmacogenomics of metabolic effects of rosiglitazone.
Seda O, Sedová L, Oliyarnyk O, Kazdová L, Krenová D, Corbeil G, Hamet P, Tremblay J, Kren V.
Pharmacogenomics. 2008 Feb;9(2):141-55. doi: 10.2217/14622416.9.2.141.
PMID: 18370844

21. Relations of **adipose tissue** CIDEA gene expression to basal metabolic rate, energy restriction, and obesity: population-based and dietary intervention studies.
Gummesson A, Jernås M, Svensson PA, Larsson I, Glad CA, Schéle E, Gripeteg L, Sjöholm K, Lystig TC, Sjöström L, Carlsson B, Fagerberg B, Carlsson LM.
J Clin Endocrinol Metab. 2007 Dec;92(12):4759-65. Epub 2007 Sep 25.
PMID: 17895319

22. Short-term resistance to **diet**-induced obesity in A/J mice is not associated with regulation of hypothalamic neuropeptides.
Bullen JW Jr, Ziotopoulou M, Ungsunan L, Misra J, Alevizos I, Kokkotou E, Maratos-Flier E, Stephanopoulos G, Mantzoros CS.
Am J Physiol Endocrinol Metab. 2004 Oct;287(4):E662-70.
PMID: 15361355

23. Segment of rat chromosome 20 regulates **diet**-induced augmentations in adiposity, glucose intolerance, and blood pressure.
Pausova Z, Sedova L, Berube J, Hamet P, Tremblay J, Dumont M, Gaudet D, Pravenec M, Kren V, Kunes J.
Hypertension. 2003 May;41(5):1047-55. Epub 2003 Mar 24.
PMID: 12654711 **Free Article**

24. BFIT, a unique acyl-CoA thioesterase induced in thermogenic **brown adipose tissue**: cloning, organization of the human gene and assessment of a potential link to obesity.
Adams SH, Chui C, Schilbach SL, Yu XX, Goddard AD, Grimaldi JC, Lee J, Dowd P, Colman S, Lewin DA.
Biochem J. 2001 Nov 15;360(Pt 1):135-42.
PMID: 11696000 **Free PMC Article**

25. Regulation of PPAR gamma gene expression by nutrition and obesity in rodents.
Vidal-Puig A, Jimenez-Liñan M, Lowell BB, Hamann A, Hu E, Spiegelman B, Flier JS, Moller DE.
J Clin Invest. 1996 Jun 1;97(11):2553-61.
PMID: 8647948 **Free PMC Article**

26. [Pharmacologic manipulation of ob expression in a dietary model of obesity.](#)
Collins S, Surwit RS.
J Biol Chem. 1996 Apr 19;271(16):9437-40.
PMID: 8621612 **Free Article**

Back to top
